# Interdisciplinary Team Collaboration during Discharge of Depressed Older Persons: A Norwegian Qualitative Implementation Study

**DOI:** 10.1155/2013/794743

**Published:** 2013-05-16

**Authors:** Anne Lise Holm, Elisabeth Severinsson

**Affiliations:** Centre for Women's, Family and Child Health, Faculty of Health Sciences, Vestfold University College, P.O. Box 2243, 3101 Tønsberg, Norway

## Abstract

In order to deliver effective care, it is necessary to organise interdisciplinary activities for older persons who suffer from depressive disorders. This paper evaluated the interdisciplinary team members' perceptions of cooperation in the discharge planning of depressed older persons based on the Chronic Care Model (CCM). A qualitative implementation design was used, data were collected by means of multistage focus groups, and a thematic analysis was performed. Three themes emerged: lack of effective team leadership in the community, the need to change the delivery system, and enhancing self-management support for depressed older persons as well as the participation of their families. It was concluded that nurse managers must find ways of supporting the depressed older persons by better structuring the care, increasing cooperation with organisational leadership, and creating an environment characterised by trust and mutual respect. Distrust can have serious implications for discharge planning collaboration. The development of a common vision of transparency in the organization is important as is a policy of change among leadership and in clinical practice.

## 1. Introduction

Depression in older persons is an increasingly complex health problem due to the difficulties involved in distinguishing it from symptoms of physical ill-health, dementia, normal aging, and grief [1, 2]. Older persons with depression are a vulnerable group for whom the transition from hospital to community care is of critical importance. Evidence has demonstrated that effective and safe interventions, delivered in collaboration between hospital and community care, are associated with a reduced rate of readmission [3]. Naylor et al. [4] identified nine interventions that demonstrated positive effects related to hospital readmission, thus constituting a key focus of health reform. According to Choi and Pak [5], interdisciplinary analysis synthesises and harmonises disciplines into a coordinated and coherent whole. Collaboration begins when different disciplines meet to share each other's knowledge [6]. Every health care professional possesses evidence-based knowledge, that is, of value to the other members of the team [7]. 

Interest in implementation issues has increased in the health services field in recent decades and can be related to the development of Evidence Based Practice (EBP) and programmes as well as the concern that people will not benefit unless EBP interventions and programmes are correctly implemented [8]. EBP requires that “decisions about healthcare are based on the best available, current, valid and relevant evidence” [9, page 1]. The CCM was presented by Wagner et al. [10, 11] in order to improve the care of persons suffering from long-term conditions such as depression. A review study by Kristofco and Lorenzi [12] found that several interventions have been developed for the purpose of enhancing the quality of depression management in primary care, including quality improvement strategies employing disease management approaches, the CCM and the Breakthrough Collaborative Series developed by the Institute of Healthcare Improvement [13]. Holm and Severinsson [14] demonstrated that a team approach aimed at enhancing care coordination led to successful implementation of the CCM. 

### 1.1. Background

An in-depth study of practices dealing with chronic conditions revealed that improved outcomes require a comprehensive system change and that most of the providers did not have the capacity to incorporate new knowledge gained from interventions [10, 11]. According to Wagner et al. [10, 11], health care systems are ineffective partly because historically they are mainly organized to respond rapidly and efficiently to acute illness. The focus on the immediate problem excluded attention to chronic conditions, and the patient's role was largely passive. If the problem is the system design, improvements cannot be achieved by further focusing on the existing system. The above authors suggested that more evidence is needed about which system changes lead to better care as well as quality improvement methods. The CCM consists of six components. (i) *Health system and organization* formed the basis of the improvement project conducted by organizational leaders and was described as a major predictor of success. Conversely, lack of leadership was a predictor of failure. (ii) *Community resources* were related to effective programming in the community through linkages with relevant agencies as a way of obtaining essential services. Negotiation with other health care organizations in the community was often important for enhancing continuity of care and expanding services. (iii) *Self-management support* included both individual and group interventions that highlighted self-management as a way of empowering different groups of persons who suffer from ill-health. The interventions were expected to help the patients to better cope with everyday life. (iv) *Delivery system design* requires planning and coordinated actions on the part of multiple caregivers, which could be performed by team members. Increasing evidence indicates the value of access to a clinical case manager, who could be an experienced nurse or pharmacist. (v) *Decision support* means that the team members need to improve their skills by means of more effective training methods. Teams must develop new relationships with relevant specialists who can provide appropriate care and educational opportunities. (vi) *Clinical information system* means a registry and database software. According to Wagner et al. [10, 11], high-quality care is characterized by productive interactions between the practice team and patients. Thus, every depressed older person needs a team led by a nurse or a general practitioner to organize and coordinate her/his care. To ensure clinical management, the team must have expertise, relevant patient information, time, and resources to act rather than just react [10, 11]. 

The coordination reform in Norway included several initiatives aimed at making the discharge from specialist to community health care more effective [15]. The reform stated that patients' need for coordinated services is not sufficiently met. The Continuity of Care Task Force of the American Society of Health System Pharmacists (ASHP) identified clinical, patient, communication, organisational, coordination, professional, policy, and technological gaps that arise after hospital discharge [16]. It is challenging to undertake initiatives to close such gaps in order to ensure continuity of care [17–19]. 

### 1.2. Aim

This paper evaluated the interdisciplinary team members' perceptions of cooperation in the discharge planning of depressed older persons based on the Chronic Care Model (CCM). 

## 2. Methods

### 2.1. Design

The project had a qualitative implementation design to illuminate the perceptions of collaboration on the part of team members and staff members from a community psychogeriatric ward. A qualitative implementation design tends to be eclectic and is based on the general premises of naturalistic inquiry [20]. 

### 2.2. Focus Group Method

Focus group interviews are widely used for exploration, confirmation, and effective collection of data pertaining to attitudes, perceptions, and opinions [21]. The discussion provides several viewpoints in a short time as the participants present their own views and respond to what is said by fellow participants [21].

In the present study, the focus group method facilitated reflection among the participants. The community team members discussed their perceptions of how collaboration in the discharge planning of depressed older persons functioned within the team as well as between the team and the psycho-geriatric ward. The CCM was implemented a month before the focus group interviews. The first author (A. L. H.) provided one and a half hours of education about the CCM to specially invited community and specialist health care staff in one community on the west coast of Norway. Information about the research project was also provided with the aim of recruiting participants for the focus group interviews. 

A month later, the researcher (A. L. H.) conducted two multistage focus group sessions together with a comoderator (A. B.), each lasting one and a half to two hours. Multistage focus groups imply that the dialogue continues for several sessions, thus increasing the possibility of developing a deeper understanding of the theme under investigation [22]. The group dynamic is created by the interaction and relationships within the group. The focus group participants were asked the following question: “As revealed in the education session, the CCM includes several concepts of importance for cooperation between community and specialist care. Can you describe the most important aspect of discharge planning for depressed older persons?” Six participants (four mental health nurses from a community team on the west coast of Norway and a nurse and a physiotherapist from a specialist psychogeriatric ward in a hospital in the same community) took part in the first focus group interview. Four of these (three mental health nurses from the community team and one member of the psychogeriatric ward team) participated in the second focus group interview.

### 2.3. Participants

The participants consisted of a four-member community team (Team A), which served adults suffering from different mental health problems as well as a geriatric nurse and a physiotherapist from the psychogeriatric ward of a hospital (Team B) in the same community. The members of Team A had all worked for a long time as mental health nurses in a range of positions in the community and specialist health care, while the two participants from Team B had had various positions in specialist health care. The physiotherapist had worked on the psychogeriatric ward for two years, while the nurse had many years' of experience working on such a ward. All the participants were women aged between 35 and 57 years. 

### 2.4. Data Analysis

The study used qualitative thematic content analysis [20, 23]. The identification of key themes was not a linear process, and the researcher had to refer to the data to check that the themes really fit and refine them where necessary. The main researcher read the transcripts, after which she reflected on, coded, and sorted the material into themes. The two researchers discussed the emerging themes until consensus was achieved. The researchers agreed on the quotations to be included to illustrate the data, their order, and the rationale for the chosen order of presentation. 

The researchers reflected on the meaning of the statements in the light of their own experiences as mental health nurses and researchers. It can be assumed that their preunderstanding influenced the description and understanding of the themes [24]. 

### 2.5. Trustworthiness

The researchers are responsible for ensuring the trustworthiness of the research process and the truthfulness of the thematic analysis. In qualitative research, the criteria for assessing the “truth value” presuppose a systematic collection of data over the entire research period [25]. According to Polit and Beck [26], the criteria for establishing the trustworthiness of qualitative data are credibility, transferability, dependability, and confirmability. Credibility refers to confidence in the truth of the data and their interpretation [26, 27]. This implied ensuring that the perspectives of the participants were represented as clearly as possible. The researchers used quotations from the text in order to help the reader to judge whether they succeeded in representing the participants' perspectives. They checked the text several times and put critical questions to it, thus enhancing the credibility of the findings. Confirmability refers to the objectivity or neutrality of the data [26, 27] and the researchers attempted to interpret the text as objectively and neutrally as possible. They read the focus group summaries several times before agreeing on the themes and subthemes. According to Lincoln and Cuba [27], this is a criterion for checking the truth value in qualitative research. Dependability refers to the systematic, logical, and documented inquiry process [20, 26] in addition to data stability over time and conditions [20, 26]. Such stability can be difficult to judge because the team members' situation may not be constant. 

### 2.6. Ethical Considerations

The study was approved by the Regional Ethics Committee of Western Norway (number 2010/2242) and carried out in accordance with the Ethical Guidelines for Nursing Research in the Nordic countries [28]. The participants were informed about the purpose and method of the study, their right to withdraw at any time [29], that the data would be treated confidentially, and that their names would be removed from the transcripts. They were asked for permission to audiotape the focus group interviews.

## 3. Results

The following three themes and seven subthemes emerged (see Table 1). The first theme, Lack of effective team leadership in the community, comprised two subthemes: the team members' obligation to report problems to their leaders and managers and lack of trust. The second theme, the need to change the delivery system, contained four subthemes: being included in transforming discharge planning, an open attitude towards what the community can offer, preventing readmission, and the need to provide better information. The third theme, Enhancing self-management support for depressed older persons as well as the participation of their families, included one subtheme: the meaning of caring.

### 3.1. Lack of Effective Team Leadership in the Community

Team A had no formal leader, and a mental health nurse acted as a clinical leader without any administrative authority. The team members believed that their function was to help persons cope with mental health problems. The mental health nurse acted as a manager in clinical matters, and the other team members had regular contact with her. However, this cooperation did not function at the next administrative level, which constituted an obstacle. One participant from Team A stated the following:

**“There is a lack of higher level leadership in the transition phase and there is no dialogue, which is really sad. We report the case to our leader, who understands it and tries to bring it to the attention of her superior, the health care leader, but the latter does not understand our needs because she is in charge of the whole health care system in the community and does not have much knowledge about the health and nursing needs of older persons with depression.” (Team A, Participant No. 1)**

Team A agreed that although the management of the home care services in its community was familiar with elder care, knowledge of older persons with depression was lacking. The team members stated that there was a need for a special team devoted to older persons who suffer from depression and that such a team requires a care manager, as recommended in the CCM, who can cooperate with the primary care physician in the event of problems. Such a model can strengthen cooperation between the team and the primary care physician, as at present, the latter has only a subordinate role in terms of cooperation with the team. 

The researcher asked which professional category was most suitable to implement such a change and whether a manager could facilitate the process. Two participants from Team A agreed that a leader has the ability to judge different initiatives and stated that documentation was vital.

**“We must include the leader.” (Team A, Participants Nos. 1 and 3)**

**“Our leaders talk while we are left sitting there with no information and can see what does not work.” (Team A, Participants Nos. 2 and 4)**

Team A agreed that there must be a dialogue about the needs of older persons with depression and that the leader has a vital role. 

#### 3.1.1. The Team Members' Obligation to Report Problems to Their Leaders and Managers

One participant from Team A raised the question of the duty to report problems to leaders and managers. “I get quite upset when I hear about things going wrong in the transition phase. In my opinion geriatric ward staff members should document problems and report them to their leader” (Team A, Participant No. 3).

The researcher asked whether someone could help them with this during the transition process. One participant from Team A stated, “it's essential that the team leader should be involved in the community administration group responsible for the implementation of the reform” (Team A, Participant No. 1). 

#### 3.1.2. Lack of Trust

The members of the community team reported that the worst aspect was the fact that the care coordination department planned discharge together with the psychogeriatric ward without contacting them, which means that they no longer had trust in nor a sense of loyalty to this department. A member of Team A stated the following:

**“I wish I could trust the community to show commitment to the development of a collaboration plan for specific patient groups such as depressed older persons.” (Team A, Participant No. 4)**

The team members stated that the professionals in the care coordination department decided what the older persons needed, prevented them from obtaining all interventions required, and mainly focused on physical needs. They added that primary care physicians did not pay much attention to depressed older persons, who reported receiving little information or assistance and felt quite lost. 

### 3.2. The Need to Change the Delivery System

The members of Teams A and B had not considered how Team A could be included in Team B's regular meetings about the discharge of depressed older persons and their various needs. The members of Team B explained that they arranged meetings with the health services administration in the older person's community without inviting members of Team A. Thus, the three members of Team A knew nothing about these depressed older persons and their needs, as it was not considered relevant. The members of Team B complained that the community health care professionals placed too much focus on administrative procedures and that a change in the delivery system was necessary. 

#### 3.2.1. Being Included in Transforming Discharge Planning

Both teams wanted to have a greater say in changing the discharge planning. They considered that the community administration's focus on lack of resources was an excuse to avoid discussing how to improve procedures.

**“When considering older persons' support needs I think that arguments such as lack of resources are very unprofessional and a way to avoid making changes. I'm quite shocked when I hear things like that.” (Team A, Participant No. 2)**

The above participant also suggested that if resources are used as an excuse for not changing the system and meeting the old persons' needs, one should make use of one's legal right to complain. A member of Team A stated the following:

**“I found this peculiar as one needs to change the organization or as it is termed in the CCM, the community health care delivery system.” (Team A, Participant No. 1)**

#### 3.2.2. An Open Attitude towards What the Community Can Offer

One of the members of Team A commented that when the administrative officer judges the situation, she/he should have an open attitude towards the way in which the team can coordinate the care of older persons, as its members have the necessary knowledge to judge the situation. She stated the following:

**“Who knows anything about depression when the team members are absent? In my opinion an open attitude towards the transition of care must include a view of what the community health care administration can offer and this should be the main issue when caring for older persons with depression.” (Team A, Participant No. 4)**

Another member of Team A stated that the team manager had been invited to some meetings, which was a solution in certain cases. One of the members of Team B agreed that while it was a good idea, it would not be a solution in all cases because members of Team A could not always attend meetings during the day. One member of Team A stated the following:

**“I object to the fact that the professionals in the administration office reject all initiatives. There must be some initiatives that can be accepted at lower level.” (Team A, Participant No. 3)**

#### 3.2.3. Preventing Readmission

One participant from Team A raised the question of how discharge could be improved in order to prevent readmission. The members of Team A agreed that older persons with depression are not eager to participate in activities. They have to be collected from their own homes and transported to various activities. As they do not participate voluntarily, psychiatric and geriatric nursing, competence, and healthcare knowledge are of the utmost importance. One member of Team B stated the following:

**“There should be special discharge procedures in place to make the transition better and safer for depressed elderly persons. Over time we can gradually eliminate any unnecessary procedures. The situation today seems to be the opposite, as the administration has increased the number of procedures to provide the minimum level of care. I think this is what leads to re-admission and is wrong in my opinion.” (Team B, Participant No. 1)**

#### 3.2.4. The Need to Provide Better Information

An issue that arose was how the teams receive information about, for example, transitions from specialist hospital care to community care as recommended by the reform initiative. One participant from Team B stated the following:

**“The somatic department of the hospital received such information, but not the geriatric or psychiatric departments.”**

The members of Team A revealed that they found it especially difficult to help the health care system disseminate information about depression and its consequences. One of them stated that the team members did not communicate with the administrative office that has the authority to plan the discharge process and often received no information about the depressed older persons. Another stated the following:

**“It is so sad and the main reason why the depressed older person does not receive better care.” (Team A, participant No. 3)**

### 3.3. Enhancing Self-Management Support for Depressed Older Persons as well as the Participation of Their Families

Team A agreed that it was wrong that the depressed elderly person lacked support when she/he was recovering after discharge. How can older persons be involved in their own discharge and encouraged to use their health resources? The members of Team A agreed that their role was to ensure the involvement and participation of depressed older persons and their families.

Two participants, one from Team A and the other from Team B, revealed that they made use of the older persons' own resources. They explained that they took the time to get to know each person, such as her/his former profession, hobbies, what she/he used to enjoy doing, and if she/he was able to continue such activities despite old age. They also agreed that it was necessary to include the family in order to obtain more information about the depressed older person. One member of Team B stated the following:

**“What have been positive life experiences? When older persons are in a depressed state they often have problems talking about self-management and how they had been in charge of their life previously. They cannot find anything positive about themselves and lack the words to express their emotional pain.” (Team B, Participant No. 2)**

Both teams agreed that the older persons seem to be solely concerned with the present; thus, family and friends can supply important information about past experiences and the future.

#### 3.3.1. The Meaning of Caring

One member of Team B was concerned about caring when the elderly person rejected contact. She revealed that her understanding of caring was taking over when the person had lost control. She considered it important to reflect on the meaning of caring based on autonomy and stated the following:

**“Older persons' level of participation is often low, thus the nurses must use their intuition in order to promote participation during the transition process.” (Team B, Participant No. 1)**

The other participant from Team B expressed the following:

**“As a professional, if rejected, one cannot withdraw from the situation based on the notion that one should not intrude. But it is often a dilemma, when is the right moment to assume control?” (Team B, Participant No. 2)**

Both teams agreed that in such situations a health care professional must know when and how to act. One participant from Team B stated the following:

**“I believe that they need help with this and that. I try my best to explain what I'm doing and why. I always attempt to negotiate with the depressed elderly person.” (Team B, Participant No. 2)**

## 4. Discussion

Perceptions of interdisciplinary team collaboration were related to the first theme, namely, that an obstacle to safe care was lack of effective team leadership in the community. The second theme revealed the need to change the delivery system, and the third that it is necessary to enhance self-management support for depressed older persons as well as the participation of their families.

*Lack of effective team leadership in the community* constituted a barrier to discharge planning. The quality and quantity of leadership support was found to be a vital component of successful discharge planning and collaboration [10, 11]. Well-functioning team leadership has been reported as important for developing communication and collaboration as well as a team culture characterised by stable, self-reinforcing work relationships [17, 18]. It is a key element in ensuring the safe and effective transfer of older persons generally between in-patient hospital care and community-based home care [3]. 

In discharge planning, the role of the team manager generally known as care manager is essential [10, 11]. Lack of clarity pertaining to the responsibility inherent in this role can constitute a challenge, as the care manager is responsible for coordinating the care of depressed older persons [11]. The care managers' roles and responsibilities require clarification in order to increase their authority, as much of their time and effort seems to be devoted to educating and motivating depressed older persons [30]. Care managers need protocols that specify their role. Such protocols are mentioned by Wagner et al. [10]. The protocols can facilitate self-management, for instance, close followup to assess the outcome of a care programme or therapy to increase self-management competence. The team members do not have any training in such procedures and can be delegated functions such as telephone followup and developing linkages with other parts of the community health care system as well as with the hospitals in order to gain experience of more complex clinical cases. 

*The team members' obligation to report problems to their leaders and managers* can be seen as a duty or moral request rather than a demand [31]. An interdisciplinary team approach can apply the ethics of caring to develop value-based roles [31] in the team and a culture in which reporting problems to leaders and managers' is valued. Authority and strength can be important when speaking on behalf of depressed older persons who are unable to speak for themselves. Thus, it seems essential for each team member to understand her/his role and responsibilities as well as those of fellow members. In addition, a shared vision of openness in the organization combined with a policy of change among the leadership and in clinical practice may be important [10]. 

*Lack of trust* was mentioned by one of the team members. Trust is defined as a positive belief in and dependence on the competency of another as well as confidence that another's actions are governed by right and moral motives [32]. However, trust can vanish if the team members experience that their leaders and managers do not listen to their problems. Distrust can develop in an environment in which there is a lack of trust. Gaining trust is described as an essential part of leadership [33]. Lack of trust can be extremely counterproductive with serious implications for discharge planning collaboration. However, the team has a responsibility to actively attempt to influence the relational process in the community. Even if collaboration does not function well, team members must continue to try to enter into a dialogue with administration, which can be challenging. The organisation can be damaged by suppressed feelings and lack of openness, which in turn can weaken the hope of better collaboration and communication in the future. A pervasive sense of justice and fairness seems to be an essential part of building trusting relationships, regardless of one's position in the community [34]. 

*Changing the delivery system* seemed to be beyond the control of the team members. However, as described in the transformational leadership literature, a culture of change is of the utmost importance when the delivery system requires change [33]. Transformational leadership can be used to understand leadership and management especially in times of organisational change. The characteristics of this form of leadership involve leaders and managers keeping their promises, behaving in line with their promises, and gaining trust [14].

*Being included in transforming discharge planning* was described as important, especially participating in meetings at which such transformation was discussed. Research has revealed the value of the care manager performing functions that promote change [10]. Each team member can delegate tasks such as telephone followup and assessment to fellow team members and use the time thus saved to develop linkages with other parts of the organisation or with specialist care in order to carry out complex clinical care managerial tasks. When planning discharge, the team can use the information register to organise visits and provide appropriate care such as supportive dialogue, therapy, and self-management programmes [10]. 

*An open attitude towards what the community can offer* can serve as a guide for transforming the community health care system as well as building a well-functioning team, thus preventing attitude and communication problems. One strategy for changing attitudes is the creation of a more effective system. Another important issue is the pathogenic perspective in which old persons are described as objects rather than independent individuals [34]. This view still seems to be prevalent today, as old persons report feeling powerless and less valued. The consequence can be that a team serving depressed older persons lacks the courage to struggle against the system. Facilitating discussion of and reflection on their own attitudes and emotions can prepare team members for having their suggestions ignored by management, while at the same time enabling them to strongly support the depressed older persons and their needs. As nurses' attitudes towards older persons seem to be problematic, leadership and management skills should focus on changing such negative attitudes. 

*Preventing Readmission.* Can increasing the collaboration between the team and hospital reduce the readmission rate? One challenge seems to be creating different strategies for supporting the older person and better structuring the care, as one-third of depressed persons are often readmitted within a few days of discharge as revealed by Desplenter et al. [19]. Effective and safe interventions delivered across the hospital-community interface have been associated with a reduction in the readmission rate [3]. The team seems to have major challenges when appointing care managers to empower depressed older persons in their everyday lives. The meaning of self-management can vary as it forms part of each person's values throughout life alongside her/his experiences of depressive ill-health. A trustworthy person is one who *listens*, can confirm and strengthen an individual's self-worth, and identity and to whom one can *tell* what it is like carrying on day by day with depressive ill health as a constant “companion.” 

*The need to provide better information* during transitions is described in the analysis. Lack of information between teams but also in the community and the hospital can result in low satisfaction on the part of professionals, team members and patients, as reported by Coleman et al. [35] and Magnusson and Lützén [36]. Thus, there is a need to ensure a better flow of information in the collaborative discharge planning. 

*Enhancing Self-Management Support for Depressed Older Persons as well as the Participation of Their Families.* What does self-management support mean? The team seems to have a policy of involving depressed older persons as active partners in the management of their everyday lives. Interventions that highlight empowerment as well as older persons' participation and involvement have been described in the literature [37]. As revealed by Cumbler et al. [38], poor social support is an element of failed transitions. Decision making during the discharge planning process is described as a right, a care management responsibility [30], and can be a way of preserving the integrity and autonomy of depressed older persons and their families. 

*The meaning of caring* is building a personal relationship that enables and empowers older persons to regain independence in the discharge process and is an essential part of the care manager's role [30]. Such relationships require openness, honesty, and valuing the older person as an individual. A reflective way of being and acting, as suggested by Tillich [39], takes account of the older persons' vulnerability and need to be in charge of their own lives. However, the findings in our study can imply forcing the depressed older person to take charge when she/he does not want to be involved. Greater awareness of the meaning of participation and self-management is necessary in order to avoid violating depressed older persons. Dignity can be threatened by the perception of being incapable of self-management. Care without dignity can lead to moral distress for team members, due to the experience of threats to dignity in the conflict between the ideal and the reality [40]. The contact established between the care manager and the depressed older person seems to indicate that the former has an important role in communicating the latter's needs to the team and the psychogeriatric ward staff. Such communication creates greater awareness of and sensitivity to older persons' loss and suffering in everyday life. In addition, team members can explore the older person's history and previous ways of managing her/his life in order to gain an insight into health beliefs and patterns. 

## 5. Recommendations and Implications

### 5.1. Nursing Practice

This study indicates that leadership is the cornerstone of interdisciplinary team practice in a health care sector undergoing radical reform [41]. The lack of clarity in health policy has been described as a challenge for those responsible for mental health teams in primary care [38]. Thus, an important task in nursing practice is to ensure better communication, information, and coordination in conjunction with discharge [42, 43] by creating an interdisciplinary team as outlined in the CCM. According to this model, a care manager (often a nurse) should coordinate the care and find ways of promoting depressed elderly persons' self-management and participation by better structuring the care during the discharge planning [18]. The care manager should cooperate with organisational leadership and create an environment of trust and mutual respect [38, 44]. A culture of silence in the health care organisation may conceal the absence of clear leadership. A care manager must cooperate with leadership at all health care system levels in order to develop a well-functioning interdisciplinary team with clear lines of authority related to the various roles.

### 5.2. Nursing Research

In this implementation study, the gap in the literature related to the need for interdisciplinary teams and collaboration with the hospital in discharge planning was described. Nursing research must deepen the knowledge of better care pathways through the health care system and learn from the CCM. Further research should be conducted at all nursing levels in order to increase nurses' understanding and knowledge of how to integrate nursing research into practice by means of greater interdisciplinary team collaboration. 

This study was conducted in a community on the west coast of Norway. The small number of participants from one interdisciplinary team makes it difficult to generalize or transfer the results to other community settings in Norway. Thus, the present findings cannot be transferred to other countries. More research is required to explore interdisciplinary team collaboration in different parts of the world. 

## 6. Conclusion

Leadership support has been found to significantly contribute to successful team collaboration in discharge planning. A shared vision of openness in the organization in addition to a policy of change among the leadership and in clinical practice is important. An organisation characterised by suppressed feelings and lack of openness can weaken the hope of better collaboration and communication in the future. In order to avoid violating depressed older persons, a team needs a leader or care manager who is aware of the meaning of participation, self-management, and the necessity of coordinating their care in an optimal way. However, more studies are needed to support these results.

## Figures and Tables

**Table 1:** Themes and subthemes that emerged in interdisciplinary team collaboration during the discharge of depressed older persons.

Lack of effective team leadership in the community	The need to change the delivery system	Enhancing self-management support for depressed older persons as well as the participation of their families
The team members' obligation to report problems to their leaders and managers	Being included in transforming discharge planning	The meaning of caring
Lack of trust	An open attitude towards what the community can offer	
	Preventing readmission	
	The need to provide better information	
